# Research of Low-Rank Representation and Discriminant Correlation Analysis for Alzheimer's Disease Diagnosis

**DOI:** 10.1155/2020/5294840

**Published:** 2020-03-19

**Authors:** Zhigang Li, Aimei Dong, Jing Zhou

**Affiliations:** ^1^School of Computer Science and Technology, Qilu University of Technology (Shandong Academy of Science), Jinan 250353, China; ^2^Key Lab of Pulp and Paper Science & Technology Ministry of Education, Qilu University of Technology (Shandong Academy of Science), Jinan 250353, China

## Abstract

As population aging is becoming more common worldwide, applying artificial intelligence into the diagnosis of Alzheimer's disease (AD) is critical to improve the diagnostic level in recent years. In early diagnosis of AD, the fusion of complementary information contained in multimodality data (e.g., magnetic resonance imaging (MRI), positron emission tomography (PET), and cerebrospinal fluid (CSF)) has obtained enormous achievement. Detecting Alzheimer's disease using multimodality data has two difficulties: (1) there exists noise information in multimodal data; (2) how to establish an effective mathematical model of the relationship between multimodal data? To this end, we proposed a method named LDF which is based on the combination of low-rank representation and discriminant correlation analysis (DCA) to fuse multimodal datasets. Specifically, the low-rank representation method is used to extract the latent features of the submodal data, so the noise information in the submodal data is removed. Then, discriminant correlation analysis is used to fuse the submodal data, so the complementary information can be fully utilized. The experimental results indicate the effectiveness of this method.

## 1. Introduction

Alzheimer's disease (AD) is a type of neurodegenerative disease, which is caused by many factors. With the increase in the aging population, the incidence and mortality of Alzheimer's disease increase year by year [1, 2]. Alzheimer's care and treatment cost up to $290 billion a year. Timely detection is the key to the treatment of Alzheimer's disease, but it is very difficult to diagnose Alzheimer's disease due to the diversity of its causes. Using medical imaging technology [3–6] to assist clinical staff in diagnosis is the primary method to detect Alzheimer's disease. Each imaging [3, 5, 6] device can display pathological information of different tissues and organs of the human body, as well as different forms of pathological information of the same organ. Because of the diversity of the causes of Alzheimer's disease and the phenomenon of brain atrophy, it is difficult to capture all the immunological information contained in the medical image only by the naked eye. So in the past decade, people began to identify Alzheimer's disease by machine learning. The computer can capture various fine-grained pathological information which cannot be obtained by human eyes. Computer-aided diagnosis [7–11] has grown up to be an important basis for clinicians to diagnose diseases. Machine learning offers a theoretical basis for it.

In recent years, the research shows that the performance of diagnosis can be substantially improved by using multimodal data with complementary information. Using multimodal data to detect Alzheimer's disease has grown up to be a research hotspot. A double-layer polynomial network [12] method is proposed. Firstly, the first-layer polynomial network extracts the high-level semantic features of MRI and PET data, and the second-layer polynomial network is employed to multimodal data fusion. This method reduces the noise of data, but will cause the loss of latent features. Zhu et al. [13] proposed a method for combining feature selection and subspace learning to identify and select features in a unified framework. Nevertheless, it ignores the internal feature structure of data. Liu et al. [14] proposed a new multitype diagnosis framework, which is composed of an automatic encoder and soft ax layers. The multimodal data are shared through the automatic encoder network spatial representation. This method can effectively learn the potential features of multimodal data, ignoring the relationship between modes. In literature [15], a new approach for HGR is proposed which is based on Quartile Deviation of Normal Distribution (QDOND) for mortal extraction and Bayesian model along with binomial distribution for features fusion and best features selection. This method does not consider the influence of noise value. In literature [16], a fusion method based on phase consistency and local Laplace energy weighting is proposed. The high-frequency and low-frequency features of diverse modal data are obtained by NSCT, in which the high-frequency features are fused by phase consistency rules and the low-frequency features are fused by local Laplace energy weighting. But the computational efficiency of this method needs to be improved.

There are for two main challenges in multimodal data fusion: (1) dealing with noise data and redundant information; (2) modeling the relationship between multimodal data effectively. The above method uses joint representation to learn the shared potential features of multimodal data. Noise information and redundant information in the data are not effectively processed, and the relationship between the multimodal data is also suppressed. In view of the above problems, we propose a feature fusion method founded on the combination of low-rank representation and discriminant correlation analysis. The proposed method has three advantages: (1) noise reduction and subspace feature learning of the original data reduce the noise value and redundancy of the data; (2) maximize the pairwise correlation between the submodal and the submodal features and effectively simulate the relationship between the modes; (3) replace the original features with fusion features to avoid noise information to the greatest extent.

The rest of the work is as follows: In Section 2, we introduce the method named LDF built on low-order representation and discriminant correlation. We further describe and review the experimental results in Section 3. Finally, we summarize this paper in Section 4.

## 2. Method

**Figure 1:**
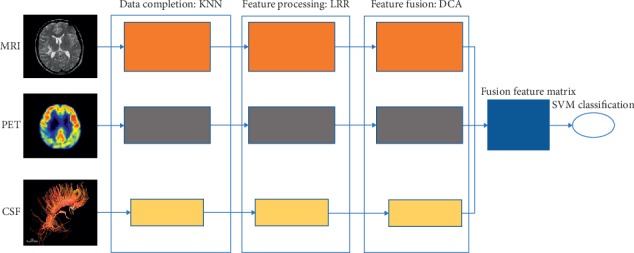
Framework of the proposed method LDF.

In this part, we will introduce the proposed method LDF in detail. The LDF method is mainly composed of four parts: data completion, feature processing, feature fusion, and SVM classification. Due to the lack of data, the KNN algorithm is used to complete the data first; secondly, low-rank representation is used to extract the potential features of the data and denoise the multimodal data; thirdly, discriminant correlation analysis is used to model the submodal data and get the fusion matrix; finally, the fusion results are input into the support vector machine classifier for classification, see Figure 1 for detail.

### 2.1. Data Completion

There are missing data in the original dataset. Existing studies [17] have shown that deleting missing data can affect the accuracy of experiments. The existing research proves that the KNN algorithm is superior to other algorithms in the supplement of missing value. At the same time, we have carried out a large number of experiments and the results demonstrated the superiority of the KNN method to other data completion methods. The core idea of the KNN [18, 19] algorithm is tantamount to the distance between the missing data items and the complete dataset, and the K value is selected closest to the missing data for weighted average, as the supplement of the true value. We choose the value of *K* as 5. The KNN algorithm replaces the missing value by finding the nearest *K* number and finding the weighted average sum of these *K* numbers:

(1) $$\begin{array}{c} \text{dist}\left(X_{i},Y_{j}\right)=\sqrt{\overset{n}{\underset{l=1}{\sum }}{\left(X_{il}-Y_{jl}\right)}^{2}},\\ \bar{x}=\frac{\sum_{i=1}^{K}wx_{ij}}{K}, \end{array}$$

where  dist(*X*_*i*_, *Y*_*j*_) is the European distance, $\;\bar{x}$ is the imputed data, and *w*=1/*d*_*i*_/∑_*i*=1_^*K*^1/*d*_*i*_  is the weight.

### 2.2. Low-Rank Representation

The original data exist in high-dimensional space and contain noise data. High-dimensional data usually contain hidden features. Low-rank representation [20–22] is a powerful and applicable tool to extract hidden features and remove noise information from high-dimensional data. Our goal is to extract latent features from high-dimensional space and remove the noise information contained in the original data. We define raw data as **A**:

(2) $$\begin{array}{c} A=\left(A_{1},A_{2},\ldots ,A_{n}\right), \end{array}$$

where **A**_1_, **A**_2_,…, **A**_**n**_ represents single submodal feature data.

The purpose of low-rank representation [20] is to determine the internal relationship between the sample points and extract a global feature. The low-rank representation of the matrix is primarily obtained through the convex optimization algorithm of gradual approximation.

In order to extract the hidden features contained in the original data and remove the noise information contained in the original data, we divide matrix **A** into two parts. The first part is a linear combination of **A** and a low-rank matrix **X** , which contains the hidden information in the original matrix. The second part is noisy data, which is a sparse matrix **E**:

(3) $$\begin{array}{c} A=AX+E. \end{array}$$

In the above formula, the solutions of **X** and **E** are infinite, but we want the solutions of **X** to be low rank, so we can convex relax the optimization problem:

(4) $$\begin{array}{c} \;\begin{array}{cc} \underset{X}{\min} & {\left\|X\right\|}_{*},\\ \text{s}.\text{t}. & \;A=AX. \end{array} \end{array}$$

We need to extract features from multiple subspaces, and in order to make noise and outliers more robust. Considering joint subspaces, they can be expressed as

(5) $$\begin{array}{c} \begin{array}{cc} \underset{X,E}{\min} & {\left\|X\right\|}_{*}+\lambda {\left|\left|E\right|\right|}_{2,1},\\ \text{s}.\text{t}. & A=AX+E, \end{array} \end{array}$$

where ||**X**||_*∗*_=∑_*i*_*σ*_*i*_ (*σ*_*i*_ is the singular value of **X**) [23], ||**E**||_2,1_ = $\sum_{j=1}^{n}\sqrt{\sum_{i=1}^{m}{\left(E_{ij}\right)}^{2}}$ is the noise regularization strategy, and *λ* is a positive free parameter, which is used to balance the weight of the low-rank matrix and the sparse matrix.

### 2.3. Feature Fusion

Discriminant correlation analysis (DCA) [24, 25] is an improved algorithm based on canonical correlation analysis (CCA). The existing feature fusion algorithm [12–15, 26] uses the neural network or sparse representation to jointly represent multimodal data, leading to suppress the relationship between multimodal data. CCA (20–22) can effectively model the relationship between multimodal data, but it cannot deal with the redundant information in the data. To this end, we propose the LDF method which uses DCA for Alzheimer's disease detection based on low-rank representation. The LDF method effectively models the relationship between submodes by maximizing the correlation of similar features. That is to say, on one hand, low-rank representation can remove the noise data existing in original data; on the other hand, DCA can minimize the correlation between different features and remove redundant information.

DCA can be divided into two parts: (1) discriminant analysis of each feature is set through the interclass scatter matrix; (2) correlation analysis between feature sets is driven by the diagonalization of the intergroup covariance matrix. The calculation formula of the scatter matrix *S*_intera_ between classes is

(6) $$\begin{array}{c} S_{{\text{intera}}_{p\times p}}=\overset{c}{\underset{j=1}{\sum }}m\left(\bar{a_{j}}-\bar{a}\right){\left(\bar{a_{j}}-\bar{a}\right)}^{T}\\ =\Phi_{\text{intera}}\Phi_{\text{intera}}^{T}, \end{array}$$

where $\Phi_{\text{intera}}=\left[\sqrt{m}\left(\bar{a_{1}}-\bar{a}\right),\sqrt{m}\left(\bar{a_{2}}-\bar{a}\right),\cdots ,\sqrt{m}\left(\bar{a_{c}}-\bar{a}\right)\right]$, $\bar{a_{j}}$ is the mean of the *j*th class and $\bar{a}$ is mean of whole feature set, and Φ_intera_Φ_intera_^*T*^ is a symmetric semidefinite matrix.

The first step of DCA is to project the feature matrix **A** into a new r-dimensional space **A**_(*r* × *n*)_′=**W**_intera_(*r* × *p*)__^*T*^**A**_(*p* × *n*)_ by finding a new transformation matrix **W**_intera_ [24]. Our aim is to reduce the correlation between different features by minimizing the correlation between different features. So we can diagonalize [27] it and change the divergence matrix *S*_intera_ between-class into *S*_intera_′=**W**_intera_^*T*^**S**_intera_**W**_intera_=Φ_intera_′Φ_intera_^′**T**^=**I**. The scattering matrix Φ_intera_′Φ_intera_^′**T**^ is a strictly diagonally dominant matrix, in which the diagonal element is close to 1 and the nondiagonal element is close to 0. The way to obtain the transformation matrix **W**_intera_ is obtained from [24]. Similarly, we can use the same method to solve for the second feature **B** to get the transformation matrix **W**_interb_ corresponding to different feature subsets, and the updated class scatter matrix *S*_interb_′=**W**_interb_^*T*^**S**_interb_**W**_*interb*_=Φ_interb_′Φ_interb_^′**T**^=**I**.

Secondly, in order to maximize the correlation between the two feature sets **A** and **B**, diagonalize the interclass covariance matrix **S**_*ab*_′=**A**′**B**′ of the transformed feature set, which is diagonalized by SVD:

(7) $$\begin{array}{c} S_{ab_{\left(r\times r\right)}=U\Sigma V^{T}}^{\prime }\Rightarrow U^{T}S_{ab_{\left(r\times r\right)}}^{\prime \prime },V=\Sigma , \end{array}$$

where Σ is the diagonal matrix made up of nonzero diagonal elements. If **W**_*ca*_=*U*Σ^−1/2^ and **W**_*cb*_=**V**Σ^−1/2^, then

(8) $$\begin{array}{c} {\left(U\Sigma^{-\left(1/2\right)}\right)}^{t}S_{ab}^{\prime }\left(V\Sigma^{-\left(1/2\right)}\right)=I. \end{array}$$

Thus, **W**_*ca*_ and **W**_*cb*_ are the transformation matrices for **A**′ and **B**′ and the resulting transformed feature sets are written as

(9) $$\begin{array}{c} A^{*}=W_{ca}^{\prime }A^{\prime }=W_{ca}^{T}W_{\text{intera}}^{T}A=W_{a}A,\\ B^{*}=W_{cb}^{\prime }B^{\prime }=W_{cb}^{T}W_{\text{interb}}^{T}B=W_{b}B. \end{array}$$

**Figure 2:**
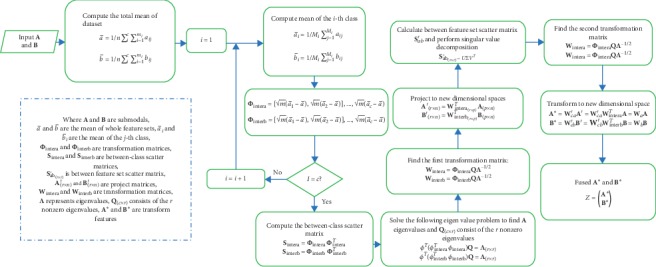
Flowchart for computing DCA-based feature-level fusion.

After getting the transformed features **A**^*∗*^ and **B**^*∗*^, we connect **A**^*∗*^ and **B**^*∗*^ in series to get the fusion matrix. The specific flowchart is shown in Figure 2.

## 3. Experiment

### 3.1. Data Set and Experimental Environment

In recent years, using multimodal incomplete heterogeneous data to detect Alzheimer's disease has become a very important clinical and research problem. The ADNI-1 database has been widely used in many studies. The ADNI-1 dataset contains the longitudinal, multisite MRI and PET image data of Alzheimer's disease, mild cognitive impairment, and elderly control patients, describing the longitudinal changes of the brain structure and metabolism, as well as clinical/cognitive and biomarker data. According to MMSE (Mini-Mental State Examination), ADNI-1 is divided into the NC (normal control) group, MCI (mild cognitive impairment) group, and AD (Alzheimer disease) group. There are 805 subjects in the baseline ADNI-1 database. Specifically, 226 subjects are NC, 393 MCI, and 186 AD. All subjects had at least one of the three data modalities: MRI, PET, and CSF. A summary of the ADNI-1 database used in this study is given in Table 1. For a detailed description of the ADNI-1 database, please visit http://adni.loni.usc.edu.

All the algorithms are carried out in Matlab2018b on a computer with Intel Core i7-8750H 2.20 GHz CPU and 8.00 GB RAM.

### 3.2. Comparison Method

Feature fusion methods are divided into the pixel-level fusion method, feature-level fusion method, and decision-level fusion method. In this experiment, we select a variety of feature-level and decision-level fusion methods to compare with our methods. The specific methods used are shown in Table 2.

Among them, KNN, SVD, and EM [24] are the three frequently used methods of data completion. KNN, SVD, and EM [24] algorithms are used to complete the missing data, and the completed data are concatenated in series to get the fusion matrix. Among them, the number of iterations of the EM algorithm is 50, and the value of K in KNN is 5. For SVD, we choose a matrix containing 95% data information. Specifically, In the KNN method, the missing data are completed with the mean of its *K*-nearest neighbor columns; in the SVD method, the missing data are iteratively computed using the matrix completion technique with low-rank approximation; in the EM method, the missing data are imputed using the EM algorithm.

CCA [28] is a traditional feature-level multimodal data fusion method, which integrates the correlation between two modes to fuse the multimodal data. Specifically, by analyzing the linear relationship between the original eigenvectors, the CCA feature-level fusion method uses the correlation criterion function to extract the typical correlation components of the two modal eigenvectors, thus obtaining the final features.

The LMP [29] algorithm uses the low-rank representation to extract the features of all the modal data and then gets the features of each modal and finally assigns different weights to these features. Parameter is the weight of modal data. For the three submodes, the different weights are 0.5, 0.25, and 0.25, respectively. Specifically, the low-rank representation is used to project the data into a low-dimensional space. The score matrix is obtained by using the relationship between the original data and the projection matrix. Different weights are assigned to different modal data according to the order of scores.

### 3.3. Analysis of Experimental Results

In this experiment, due to the missing of data, we used the KNN algorithm to complete the data. We obtained a *K* value of 5 (different from the value of *K* in the comparison algorithm). We used 10-fold cross-validation strategy to evaluate all comparison methods. Specifically, we first randomly partitioned the whole dataset into 10 subsets, and then selected one subset for testing and used the remaining 9 subsets for training. We repeated the whole process 10 times to avoid the possible bias in dataset partitioning during cross-validation, and then the averaging result was adopted as the final result.

**Figure 3:**
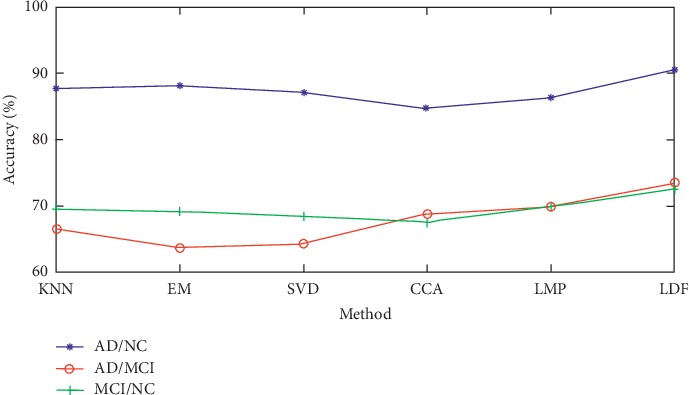
Classification result curves of six different methods of three classification tasks.

We performed extensive experiments with the datasets demonstrated in Table 1 and the parameter settings demonstrated in Table 2. The obtained results are reported in Tables 3–5 and Figures 2 and 3. In this experiment, we selected ACC (accuracy), SEN (sensitivity), SPE (specificity), BAC (balanced accuracy), PPV (positive predictive value), NPV (negative predictive value), and other indicators as the evaluation criteria to compare our method with other methods. According to these indicators, we can know the prevalence, missed diagnosis rate, and misdiagnosis rate of our method. We choose the average value and standard deviation of each index result in ten experiments as the final output. Due to the limited space, in this paper, we only choose the ROC curve for MCI/NC and the contrast experiment of time complexity, and the ROC curves for AD/NC and MCI/AD are similar to that of MCI/NC.x

Table 3 shows our experimental results of AD/NC. From Table 3, we can see clearly that our method has achieved good results in all aspects compared with other methods. Compared with other methods, our method has improved the accuracy by about 3.5%. At the same time, our method has performed well in sensitivity. Compared with other methods, our method has improved the accuracy by about 6%, and in NPV and BAC by about 5%, which shows that our method can accurately identify Alzheimer's patients.

It is difficult to find Alzheimer's disease in the early stage. Timely discovery is the best way to treat Alzheimer's disease. From Table 4, we can see that our method can more accurately detect the early symptoms of Alzheimer's disease. Our method has made good achievements in this respect. Compared with other methods, our method has improved the accuracy by 5%, the sensitivity by 25% compared with several feature-level fusion methods, and the BAC by 25% compared with other methods The increase of 5% indicates that our method can diagnose mild cognitive impairment more accurately.

Table 5 shows the experimental results of AD/MCI. Compared with other methods, our method has improved the accuracy by about 4%. We can see the effectiveness of our method through the experimental results of AD/NC, AD/MCI, and MCI/NC.

In this experiment, the time complexity of several methods is also analyzed and compared. The results are shown in Table 6. Compared with other methods, our method needs more time. In the future work, we will further optimize it to reduce the time complexity.

In order to analyze the experimental results more clearly, we have carried out visual processing on the experimental results, as shown in Figure 3. In Figure 3, we can clearly see that in this experiment, compared with the decision-making level fusion method, the feature and fusion methods get better results in terms of accuracy, and our method also gets better results in terms of accuracy.

**Figure 4:**
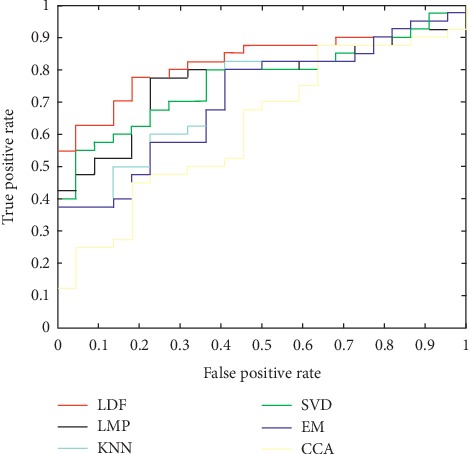
ROC curves of six different methods of MCI/NC classification tasks.

Figure 4 shows ROC curves of several methods. It can be clearly seen from the figure that our method can achieve more accurate results than other methods. Through the area under the curve (AUC), we can see that our method is obviously superior to other methods. At the same time, our method is better than other methods in disease recognition.

## 4. Conclusions

In this paper, we propose a feature fusion method based on low-rank representation and discriminant correlation analysis (LDF). Firstly, we use low-rank representation to extract the features of the original data and then use DCA to fuse the features. The experimental results show that our results are effective. In the future work, we will continue to improve our method. While modeling the relationship between modes, we will ensure that the relationship between contexts within modes will not be affected. At the same time, we will continue to improve and simplify it to obtain good time complexity in case of big data application.

## Figures and Tables

**Table 1:** Summary of the adopted datasets.

	MRI	PET	CSF	Total
Number of features	93	93	3	189
AD subjects	186	93	102	51
MCI subjects	393	201	190	97
NC subjects	226	101	112	52
Total subjects	805	395	404	200

**Table 2:** Description of the compared method.

	Comparison method	Parameter selection
Feature level	EM	50
	KNN	7
	SVD	95%
	CCA	None

Decision level	LRRF	0.5; 0.25; 0.25

**Table 3:** Classification results achieved by 6 different methods for the classification task AD/NC.

	ACC	SEN	SPE	BAC	PPV	NPV
KNN	87.83 ± 3.71	91.47 ± 5.13	85.60 ± 7.38	88.54 ± 2.79	89.95 ± 5.94	88.60 ± 4.73
EM	88.09 ± 4.36	83.96 ± 8.05	91.32 ± 4.11	87.64 ± 4.33	88.35 ± 5.42	88.16 ± 7.05
SVD	87.07 ± 5.52	88.31 ± 6.19	**94.73** ± **4.63**	83.18 ± 10.70	**91.31** ± **8.18**	86.08 ± 7.09
CCA	84.71 ± 3.93	89.04 ± 7.16	79.85 ± 5.28	84.44 ± 4.17	83.96 ± 3.10	86.06 ± 9.48
LMP	86.31 ± 5.62	82.74 ± 8.32	93.19 ± 3.28	87.97 ± 4.65	91.26 ± 4.00	86.36 ± 6.99
LDF	**90.52** ± **3.69**	**94.79** ± **3.98**	85.92 ± 7.67	**91.35** ± **4.15**	88.72 ± 7.52	**93.17** ± **4.99**

**Table 4:** Classification results achieved by 6 different methods for the classification task MCI/NC.

	ACC	SEN	SPE	BAC	PPV	NPV
KNN	66.53 ± 4.19	8.28 ± 5.19	**97.58** ± **2.63**	53.49 ± 2.24	65.69 ± 4.78	**82.72** ± **19.24**
EM	63.71 ± 8.34	25.60 ± 9.27	86.46 ± 5.40	56.03 ± 4.39	52.93 ± 12.35	66.05 ± 10.13
SVD	64.34 ± 5.67	26.01 ± 8.90	85.19 ± 8.47	55.59 ± 7.28	49.74 ± 20.72	68.31 ± 5.21
CCA	68.78 ± 4.94	25.63 ± 8.12	91.66 ± 7.68	58.65 ± 4.17	69.95 ± 4.59	68.52 ± 23.53
LMP	69.90 ± 5.63	**55.98** ± **11.79**	77.11 ± 7.70	66.54 ± 6.66	**73.99** ± **8.15**	59.04 ± 16.56
LDF	**73.49** ± **5.36**	48.64 ± 10.30	86.28 ± 6.50	**67.76** ± **5.38**	67.59 ± 13.20	76.80 ± 7.07

**Table 5:** Classification results achieved by 6 different methods for the classification task AD/MCI.

	ACC	SEN	SPE	BAC	PPV	NPV
KNN	69.48 ± 5.14	97.65 ± 2.56	13.87 ± 7.11	55.76 ± 3.61	69.13 ± 5.09	**80.00** ± **20.11**
EM	69.14 ± 3.94	12.67 ± 4.55	**98.51** ± **2.09**	55.61 ± 3.16	**80.83** ± **27.79**	68.40 ± 4.66
SVD	68.45 ± 4.81	19.18 ± 12.60	93.91 ± 5.00	56.57 ± 5.70	65.39 ± 30.62	68.91 ± 4.69
CCA	67.55 ± 4.93	**98.12** ± **2.25**	3.88 ± 3.70	52.43 ± 4.19	67.79 ± 5.23	59.52 ± 34.50
LMP	69.96 ± 6.62	93.98 ± 3.69	26.36 ± 8.87	**59.97** ± **5.15**	70.13 ± 7.23	70.17 ± 17.99
LDF	**72.54** ± **5.78**	16.46 ± 6.40	96.45 ± 2.84	56.45 ± 4.13	66.17 ± 25.34	73.16 ± 6.59

**Table 6:** Running time achieved by 6 different methods for the classification task MCI/NC.

	KNN (s)	EM (s)	SVD (s)	CCA (s)	LMP (s)	LDF (s)
Running time	3.256	4.362	3.358	0.254	10.508	5.66

## Data Availability

All the data used in the manuscript can be downloaded from the ADNI website at http://adni.loni.usc.edu/.
